# Brown Tumor From Secondary Hyperparathyroidism Mimicking Metastatic Renal Cell Carcinoma in a Patient With End-Stage Renal Disease

**DOI:** 10.7759/cureus.59376

**Published:** 2024-04-30

**Authors:** Leslie Fogwe, Venu M Ganipisetti, Kushal Naha

**Affiliations:** 1 Internal Medicine, University of Missouri School of Medicine, Columbia, USA; 2 Hospital Medicine, Baystate Medical Center, Springfield, USA; 3 Hematology and Medical Oncology, University of Missouri- Columbia, Columbia, USA

**Keywords:** end-stage renal disease (esrd), brown tumor, bone lesion, renal cell carcinoma (rcc), hyperparathyroid

## Abstract

Brown tumors (also known as osteitis fibrosa cystica) are rare complications of end-stage renal disease (ESRD) and secondary hyperparathyroidism (HPT), characterized by focal bone lesions that resemble neoplasms. They are often misdiagnosed as metastatic bone disease, especially in patients with a history of malignancy. We present a case of a 60-year-old man with a history of renal cell carcinoma (RCC), and ESRD on hemodialysis (HD), who developed diffuse bone lesions on imaging with osteolytic/osteoblastic appearance concerning metastases, but on further workup was found to have brown tumors. We discuss the treatment and outcome and briefly review the relevant medical literature.

## Introduction

Brown tumors (also known as osteitis fibrosa cystica (OFC)) are rare complications of end-stage renal disease (ESRD) and secondary hyperparathyroidism (HPT), characterized by focal/multifocal bone lesions that resemble neoplasms. They are often misdiagnosed as metastatic bone disease, especially in patients with a history of malignancy. Metastatic renal cell carcinoma (RCC) can have an aggressive course and can present with synchronous or metachronous metastasis in up to 20-30% of patients [1]. The 5-year overall survival of mRCC is about 20%. The most common sites of metastasis include the lungs, bones, liver, lymph nodes, adrenal glands, and brain. RCC has a predilection for involvement of the skeleton, and bone metastases can be seen in 20-50% of patients with RCC [2]. This case describes a 60-year-old male with a complex medical history, including ESRD with hemodialysis (HD) with HPT and RCC treated with a nephrectomy who presented with multiple bone osteolytic/osteoblastic lesions and was initially felt to have metastatic disease but was subsequently diagnosed with brown tumors. Additionally, we will discuss the pathogenesis, diagnosis, prognosis, and management of brown tumors.

## Case presentation

A 60-year-old male was admitted to the emergency department (ED) for right lower extremity cellulitis. His blood cultures grew Pseudomonas, and he was treated with a two-week course of cefepime and vancomycin. He returned to the ED one month later with persistent pain in his left upper extremity but without any signs of systemic infection. Of note, the patient denied any prior injuries or trauma, numbness or tingling in his hands, fever, or chills. He reported swelling of the left wrist and focal pain which was worsened with movement. The patient had an extensive past medical history of atrial fibrillation on amiodarone, hypertension, gout, and ESRD secondary to occult vesicoureteral reflux complicated by severe pyelonephritis requiring left nephrectomy six years ago, prior to left living donor renal transplant (LDKT) about 30 years ago, with graft failure 23 years later due to renal graft artery stenosis. Additionally, he had undergone a right nephrectomy five years ago for a renal mass that was subsequently confirmed to be RCC, Fuhrman grade 2 with chromophobe-type histology. The surgical margins were negative, and the pathologic stage was T1a [3]. The patient was already on renal replacement therapy with HD when he was diagnosed with RCC and was being reevaluated for a repeat renal transplant as it had been over 20 years since his initial transplant. He was receiving sevelamer 800 mg three times daily for hyperphosphatemia and chronic kidney disease-related mineral bone disease (CKD-MBD). Additionally, he was on amlodipine, carvedilol, and hydralazine for hypertension, and allopurinol for gout.

On physical examination, the patient had tenderness in his left forearm over the ulnar bone. Motor and sensory function was intact, and he had palpable pulses in the left upper extremity. Laboratory investigations revealed leukopenia with a WBC count of 1.90 x 10^9^/L (normal range: 4.8-10.8 x 10^9^/L), macrocytic anemia with a hemoglobin of 7.3 g/dL (normal range: 13.6-18.0 g/dL) and a mean corpuscular volume (MCV) of 100.4 fL (normal range: 80-100 fL), normal folate and vitamin B12 levels (24 ng/mL, and 426 pg/mL respectively; normal range 4.6-34.8 ng/mL, 232-1245 pg/mL, respectively), serum creatinine of 4.70 mg/dL (normal range: 0.7-1.2 mg/dL), with an epidermal growth factor receptor (EGFR) of 14 mL/min/1.7 m^2^ (normal: >=90), severe secondary HPT with a parathyroid hormone (PTH) level of 1642 pg/mL (normal range: 15.0-65.0 pg/mL), a serum calcium of 9.6 mg/dL (normal range: 8.6-10.2 mg/dL), a phosphate level of 3.9 mg/dL (normal range: 2.5-4.5 mg/dL), an elevated alkaline phosphatase of 288 units/L (normal range: 40-129 units/L) indicative of rapid bone turnover and finally a magnesium level of 1.7 mg/dL (normal range: 1.7-2.6 mg/dL).

**Figure 1:**
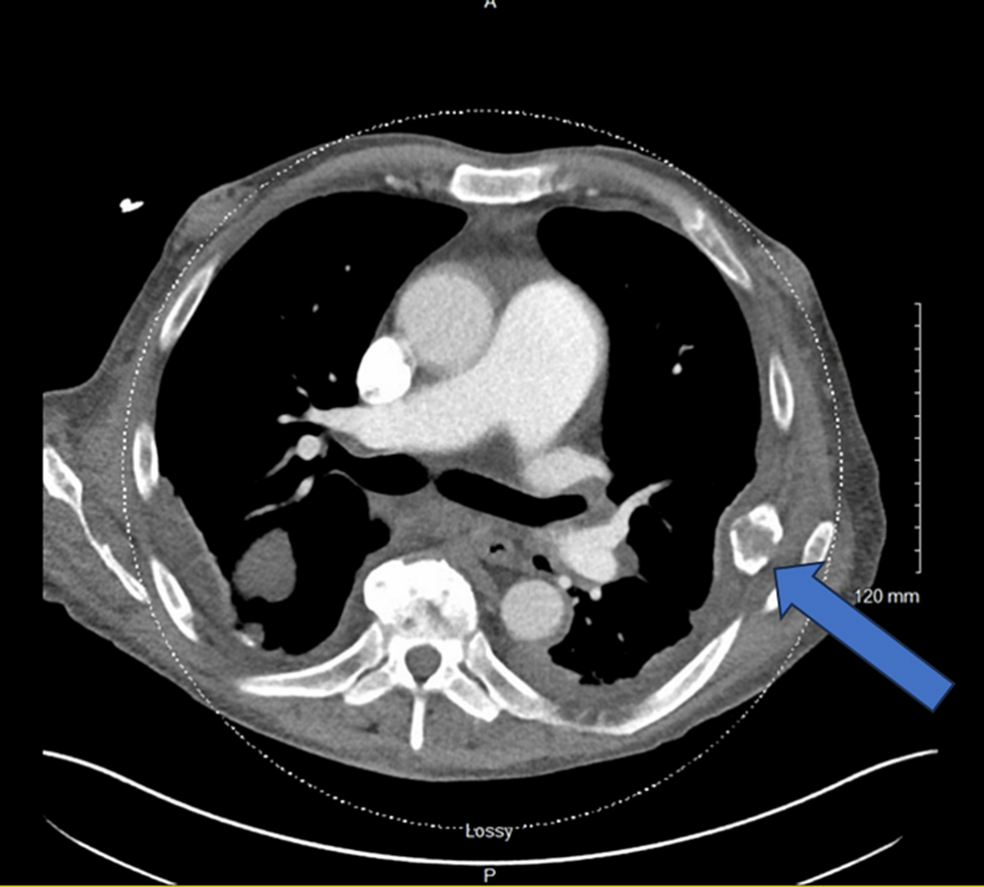
CT chest showing left sixth rib lytic lesion.

**Figure 2:**
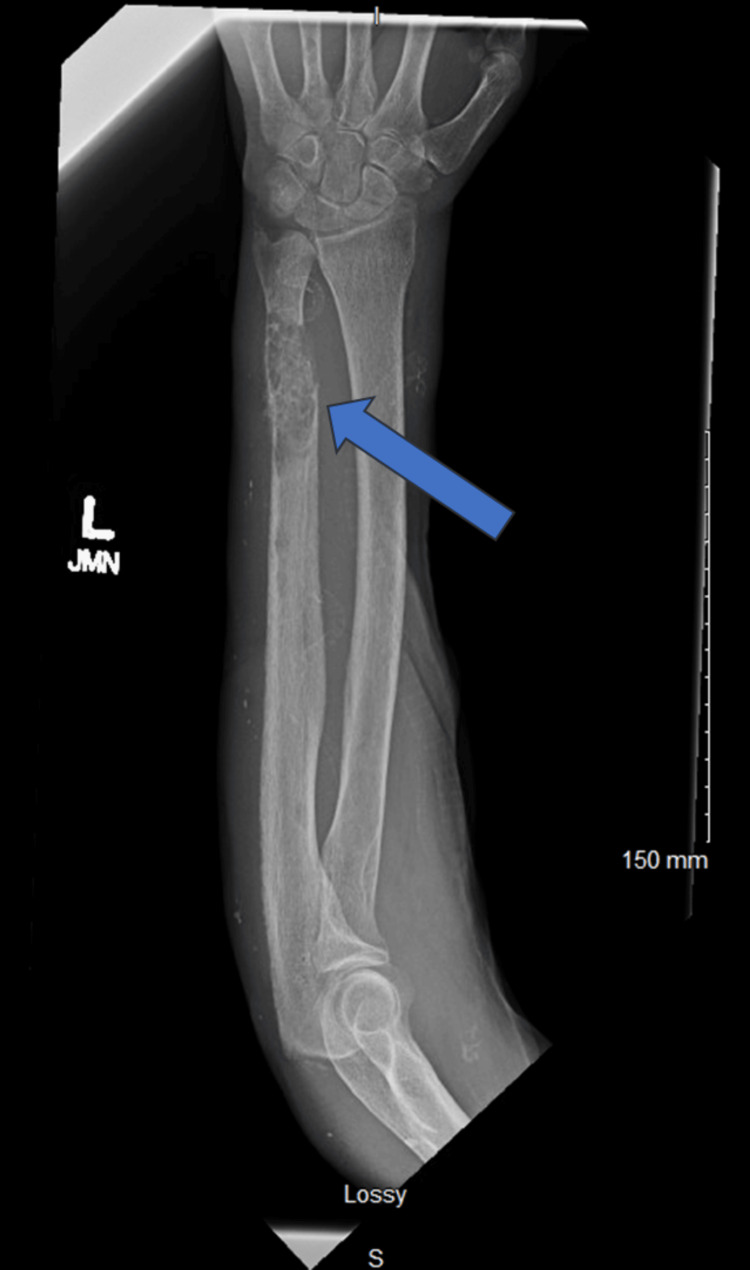
X-ray showing left distal ulnar lytic lesion.

Computed tomography (CT) of the chest, abdomen, and pelvis, performed for routine pretransplant evaluation demonstrated multiple lytic lesions concerning metastases including the left sixth rib (Figure 1). Additional evaluation of the extremities with X-rays demonstrated a bony lesion in the head of the left ulna (Figure 2).

**Figure 3:**
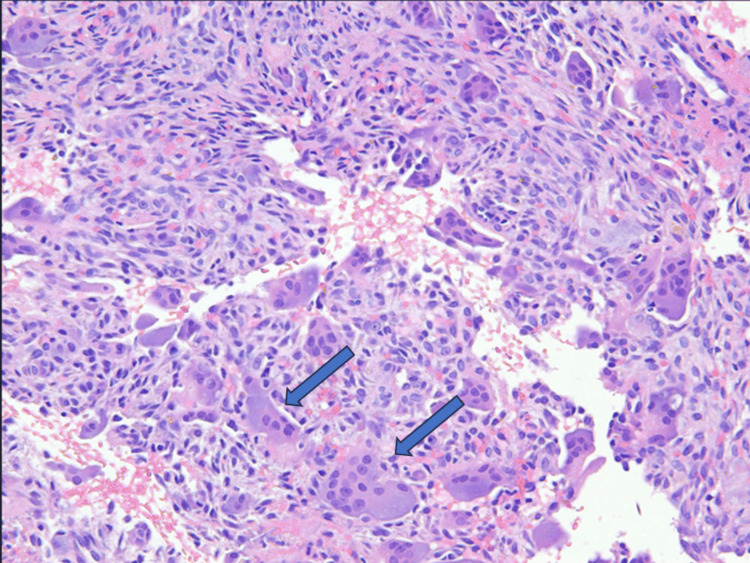
Photomicrograph of bone biopsy specimen showing numerous multinucleated giant cells (arrows) and no evidence of malignancy.

Evaluation for multiple myeloma (MM) with serum and urine electrophoresis returned negative. He was evaluated by orthopedic oncology and underwent a percutaneous bone biopsy. Histopathology showed abundant blood clots with minor amounts of admixed benign bone and bone marrow, numerous multinucleated giant cells consistent with osteoclasts, and no evidence of malignancy (Figure 3). A final diagnosis of brown tumor of HPT was made. 

The dose of PTH levels were checked and remained grossly elevated at 1315 pg/mL. His serum calcium was noted to be normal to high and his phosphorus levels were normal to low. A final diagnosis of severe secondary HPT with possible conversion to tertiary HPT was made, and he was started on cinacalcet 90 mg daily and calcitriol 3 mcg three times a week. Additionally, the dose of sevelamer was increased to 1600 mg three times daily for CKD-MBD. He was evaluated by Nephrology in the clinic and referred to Otolaryngology for parathyroidectomy.

## Discussion

Brown tumors are focal lesions seen in the axial skeleton as a result of bone remodeling because of high osteoclastic bone turnover from either persistent HPT or paraneoplastic syndrome with elevated parathyroid hormone-related peptide (PTH-rP) [4-5]. The incidence of bone tumors is 3% and 1.5% in primary and secondary HPT, respectively [4]. They are most often seen with primary HPT from parathyroid adenoma in the fifth and sixth decades of life but can also be seen less commonly in secondary HPT with ESRD. Primary HPT leads to increased serum calcium and reduced serum phosphate, in contrast to secondary HPT, where decreased renal phosphate clearance leads to elevated serum phosphate. In turn, excess serum phosphate binds to free serum calcium, leading to hypocalcemia. Ultimately, this drives the constant production of PTH through a positive feedback loop in an attempt to restore calcium homeostasis [4]. This was likely the mechanism of secondary HPT seen in our patient.

Brown tumors present as slow-growing palpable bony swellings, which may cause bone pain and pathologic fractures and may even be misdiagnosed as cellulitis, as was the case in our patient [4]. Patients can also present with symptoms of hypercalcemia in HPT, including weight loss, polyuria, and weakness, which can also be seen in humoral hypercalcemia of malignancy (HHM). Interestingly, bone pain in brown tumors is comparatively less severe than the corresponding radiographic findings suggest. Hence, these lesions are often incidental findings on radiographs or CTs performed for other indications. Dedicated imaging with CT, MRI, and sometimes positron emission tomography (PET/CT) are often performed to assess the full extent of skeletal involvement and to guide biopsy of the lesions [4]. Brown tumors typically present as single or multiple well-defined osteolytic lesions, usually with bone expansion, bony destruction, and associated pathologic fractures, and can occur in any part of the axial skeleton, often mimicking metastatic tumors [5]. Similarly, skeletal metastases from RCC are typically osteolytic and occur predominantly in the axial skeleton. They frequently lead to skeletal-related events such as pathological fractures, which reduce the quality of life and carry a worse prognosis [6-7]. However, brown tumors can demonstrate significant variability in their appearance, and malignancy should be ruled out by tissue sampling in patients with ill-defined lesions, mixed lytic/sclerotic lesions, or involvement of adjacent soft tissue [4]. The histology of brown tumors consists of clusters of osteoclasts mixed with fibrous tissue (fibroblast), poorly mineralized woven bone, and brown discoloration from hemosiderin deposition. Given its presentation with multinucleated giant cell-containing lesions, correlation with hypercalcemia and HPT is essential for differentiation between brown tumors and other giant cell tumors [4].

A key tenet in the management of brown tumors is the reversal of HPT, either by removal of the parathyroid glands in case of primary HPT with parathyroid adenoma or correction of underlying pathology driving HPT in the case of secondary HPT, especially in the setting of ESRD on HD [5]. This strategy includes vitamin D analog replacement, especially calcitriol, and calcimimetics, such as cinacalcet, to stifle the effects of PTH on peripheral bone resorption. It is also important to control hypercalcemia and hyperphosphatemia with non-calcium-containing binders such as sevelamer and lanthanum and calcium-containing phosphate binders such as calcium citrate, acetate, and carbonate (in the presence of severe symptomatic hypocalcemia). Brown tumors often regress spontaneously with reversal of HPT [5]. However, in cases of refractory HPT with persistently elevated PTH levels > 800 pg/mL if symptomatic and > 1000 pg/mL if asymptomatic, despite maximal reversal/replacement therapy, surgical parathyroidectomy should be considered. Surgery should also be considered for refractory HPT in patients awaiting transplantation, such as our patients. Oncologic resections are never indicated for brown tumors as they do not have malignant potential. Obtaining bone biopsy for concerning lesions in the setting of persistently elevated PTH levels and ruling out giant cell tumors and MM with histologic and biochemical workup should help astute surgeons avoid unnecessary surgery for these patients.

Surgical stabilization or open fixation for brown tumors is typically not needed but may be indicated in rare cases of pathologic fractures, impending pathologic fractures of major weight-bearing joints, large tumors > 2 cm cortical involvement of long bones, or lytic destruction of > 50% width of bone [8].

Brown tumors are relatively rare in patients with ESRD on HD with secondary HPT. They can present as mixed lytic/blastic lesions synchronously or metachronously in the axial skeleton, which can often be confused for metastatic disease, especially in patients with a prior history of malignancy. A characteristic histologic finding of multinucleated giant cells with osteoblasts and hemosiderin blood clot degradation products with persistently elevated PTH levels > 800 can effectively confirm this diagnosis. Management of brown tumors in ESRD patients on HD is centered on lowering PTH levels through the repletion of vitamin D, management of hyperphosphatemia, surgical parathyroidectomy as well, and surgical stabilization/fixation of pathologic fractures in rare cases. Further research is needed to determine the optimal surgical management of benign brown tumors with pathologic fractures.

Timely diagnosis of mRCC is crucial for initiating tailored therapy and prognostication. In this case, the patient’s history of RCC raised concern for metastasis despite negative tumor margins on his initial pathology. Although this patient had a diagnosis of ESRD on HD and recorded hypercalcemia and hyperphosphatemia, he did not have an established diagnosis of HPT, resulting in extensive evaluation for potential metastatic disease.

Chromophobe RCC is a distinct subtype of RCC accounting for 5% of all renal neoplasms [9] and is often associated with hereditary autosomal dominant Birt-Hogg-Dube syndrome from germline mutation in the folliculin gene [9]. Surgical resection is recommended for localized tumors either with nephron-sparing (partial nephrectomy) when feasible or radical nephrectomy [9]. Although nephrectomy is curative for patients presenting with localized tumors, such as our patient, approximately 1/3 of patients will develop late metastasis [10], which must be considered in the differential of bone lesions. Indeed, bone is the second most common distant site of metastasis after the lungs, and is seen in 20-30% of all cases of mRCC [6,10]. Risk factors for skeletal metastases include large tumor size T>/= 3, sex male > female, N stage >/=1, grade, and histologic type [6].

Bony metastases are a close differential of brown tumors due to their similar radiographic features and should be considered as a differential diagnosis in appropriate settings. In this case, the history of renal cancer was a compelling reason to consider bone metastases as a differential. However, bone metastases can arise from a broad range of common cancers including but not limited to lung cancer, prostate cancer, breast cancer, and bladder cancer, all of which may be distinguished by histopathologic sampling. Secondary tumors of the bone such as these metastases are also far more common than primary bone tumors, although these may also be considered in the differential diagnosis of brown tumors. The vastly different prognoses and treatment strategies for these conditions underline the importance of accurately distinguishing between these diagnostic possibilities.

## Conclusions

In conclusion, brown tumors represent a unique pathological manifestation primarily associated with HPT and are characterized by distinctive osteolytic lesions due to elevated osteoclastic activity. The clinical presentation can be deceptive, often resembling even malignancies like metastatic tumors, leading to diagnostic challenges. To effectively manage this condition, accurate differentiation of brown tumors from other giant cell tumors and malignant entities must be achieved through detailed histological examination, imaging studies, and analysis of the patient's biochemical profile. Although there are no established guidelines for approaching these cases, the diagnosis is typically made by biopsy confirmation rather than relying on the radiographic appearance alone. Biopsy confirmation should be considered even when a history of cancer is absent as many tumors can have bone involvement at first presentation and the bone lesions themselves are often more obvious and more symptomatic than the primary tumor site. A cursory evaluation for cancer in such cases can potentially miss the diagnosis which can have catastrophic consequences for patients.

Management strategies focus primarily on addressing the underlying HPT. Surgical intervention is crucial in cases of primary HPT, while medical management is preferred in secondary HPT related to conditions like ESRD. Ultimately, a multidisciplinary approach involving endocrinologists, nephrologists, and surgeons is essential to optimize outcomes, prevent unnecessary interventions, and ensure that patients receive treatments appropriate to the underlying etiology.

## References

[1] Matuszczak M, Kiljańczyk A, Salagierski M (2023). Surgical approach in metastatic renal cell carcinoma: A literature review. Cancers.

[2] Fottner A, Szalantzy M, Wirthmann L, Stähler M, Baur-Melnyk A, Jansson V, Dürr HR (2010). Bone metastases from renal cell carcinoma: Patient survival after surgical treatment. BMC Musculoskelet Disord.

[3] Delahunt B, Eble JN, Samaratunga H, Thunders M, Yaxley JW, Egevad L (2021). Staging of renal cell carcinoma: Current progress and potential advances. Pathology.

[4] Xie C, Tsakok M, Taylor N, Partington K (2019). Imaging of brown tumours: A pictorial review. Insights Imaging.

[5] Zhong Y, Huang Y, Luo J, Ye Y (2022). Misdiagnosis of brown tumour caused by primary hyperparathyroidism: A case report with literature review. BMC Endocr Disord.

[6] Fan Z, Huang Z, Huang X (2021). Bone metastasis in renal cell carcinoma patients: Risk and prognostic factors and nomograms. J Oncol.

[7] Zekri J, Ahmed N, Coleman RE, Hancock BW (2001). The skeletal metastatic complications of renal cell carcinoma. Int J Oncol.

[8] Fidler M (1981). Incidence of fracture through metastases in long bones. Acta Orthop Scand.

[9] Petejova N, Martinek A (2016). Renal cell carcinoma: Review of etiology, pathophysiology and risk factors. Biomed Pap Med Fac Univ Palacky Olomouc Czech Repub.

[10] Wood SL, Brown JE (2012). Skeletal metastasis in renal cell carcinoma: Current and future management options. Cancer Treat Rev.

